# Gene Regulation of Biofilm-Associated Functional Amyloids

**DOI:** 10.3390/pathogens10040490

**Published:** 2021-04-19

**Authors:** Khushal Khambhati, Jaykumar Patel, Vijaylaxmi Saxena, Parvathy A, Neha Jain

**Affiliations:** Department of Bioscience and Bioengineering, Indian Institute of Technology Jodhpur NH 65, Nagaur Road, Karwar, Rajasthan 342037, India; khambhati.1@iitj.ac.in (K.K.); patel.12@iitj.ac.in (J.P.); vijaylaxmis@iitj.ac.in (V.S.); parvathy.1@iitj.ac.in (P.A.)

**Keywords:** biofilms, functional amyloids, gene regulation, extracellular matrix, CsgA, TasA, PSM

## Abstract

Biofilms are bacterial communities encased in a rigid yet dynamic extracellular matrix. The sociobiology of bacterial communities within a biofilm is astonishing, with environmental factors playing a crucial role in determining the switch from planktonic to a sessile form of life. The mechanism of biofilm biogenesis is an intriguingly complex phenomenon governed by the tight regulation of expression of various biofilm-matrix components. One of the major constituents of the biofilm matrix is proteinaceous polymers called amyloids. Since the discovery, the significance of biofilm-associated amyloids in adhesion, aggregation, protection, and infection development has been much appreciated. The amyloid expression and assembly is regulated spatio-temporarily within the bacterial cells to perform a diverse function. This review provides a comprehensive account of the genetic regulation associated with the expression of amyloids in bacteria. The stringent control ensures optimal utilization of amyloid scaffold during biofilm biogenesis. We conclude the review by summarizing environmental factors influencing the expression and regulation of amyloids.

## 1. Introduction 

Biofilm is an aggregative behavior of microbial cells for self-protection and better survival [1]. Microbes within a biofilm can cooperate and coordinate with each other allowing them to adopt a community-based lifestyle [2]. Initially, marine microbiologists used the term “biofilm” to distinguish planktonic and adherent bacterial cells, but sooner, it was recognized as a major concern for both environment and human health [3]. Bacterial colony within a biofilm is encapsulated by an extracellular matrix (ECM) that aids in substrate adhesion [2] and acts as a stronghold for microbial cells against environmental insults [1]. It facilitates water retention, absorption of inorganic ions and organic compounds, excess carbon storage, horizontal gene transfer, polymicrobial interaction, mechanical stability, antibiotic resistance, and biofilm architecture development [1,4,5,6]. Macromolecules, such as extracellular DNA (eDNA), polysaccharides, and proteins are the key components of the biofilm matrix [4]. The proteinaceous bacterial ECM components consist of pili, flagella, adhesins, secreted extracellular proteins, and proteins of outer membrane vesicles [7].

One of the secreted ECM proteinaceous components of a bacterial biofilm matrix is amyloids [8]. Amyloids are structured protein aggregates initially known to be associated exclusively with the pathological manifestation of human diseases [9]. However, the notion changed with the attribution of functional roles to the amyloids [10]. Both pathological and functional amyloids share similar biophysical and biochemical features [10,11,12]. Functional amyloids are ubiquitous and play a pivotal role in biofilm development, growth of aerial structures, modulation of melanin synthesis, scaffolding, epigenetic control of polyamines, and transmission of genetic information [12,13].

**Figure 1:**
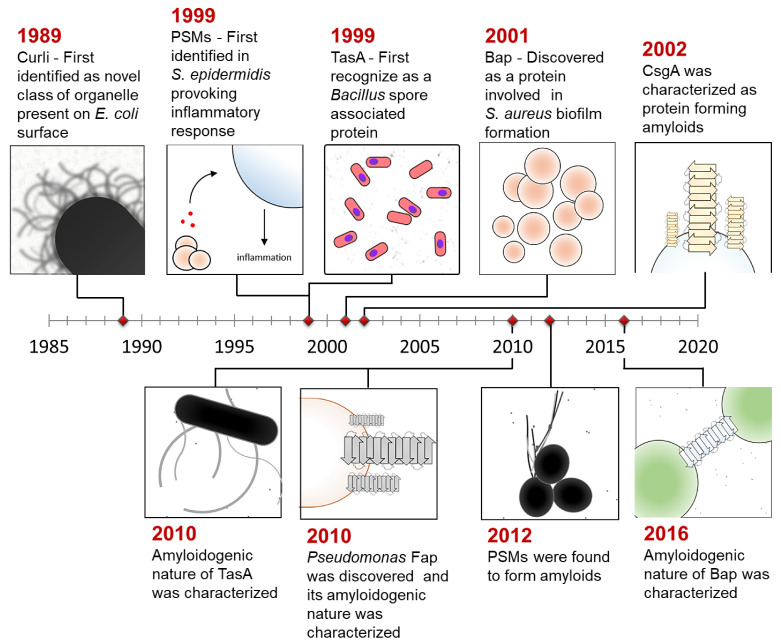
Timeline representing the discovery of functional amyloids.

The amyloid scaffold is a well-suited structure for biofilm assembly since it provides rigidity and stiffness to the matrix [14]. Figure 1 summarizes the discovery of biofilm-associated amyloids in *Escherichia coli* (CsgA and CsgB; **c**urli **s**pecific **g**ene) [15], *Pseudomonas aeruginosa* (Fap) [16], *Bacillus subtilis* (TasA and TapA) [17], and *Staphylococcus aureus* (**p**henol **s**oluble **m**odulins: PSMs and **b**iofilm-**a**ssociated **p**roteins: Bap) [18,19]. As a part of the biofilm matrix, amyloids contribute to adhesion onto the abiotic and biotic surface, increase hydrophobicity and promote colonization [20]. They also increase structural stability, provide resistance against environmental stresses, drive protection against phage particles and matrix-degrading enzymes [21]. Apart from functional roles, bacterial amyloids are also associated with disease pathology and are known to enhance gut inflammation, provoke host cytolysis, and influence neuronal-inflammation and cerebral amyloid aggregation [22,23]. Some of them are recognized as microbial-associated molecular patterns (MAMPs) and help evoke host immune response [24]. Overall, functional amyloids play a major role in biofilm formation and contribute to disease progression. Therefore, it is essential to understand the regulatory mechanism that controls amyloid assembly during biofilm development. 

In this review, we discussed the genetic regulation that controls the expression of functional amyloids in *E. coli, B. subtilis,* and *S. aureus*. As a prelude, we briefly described biofilm formation and its genetic regulation, followed by a detailed description of the gene regulation of amyloids associated with biofilm formation. The description of genetic regulation of all the matrix components of biofilm is beyond the scope of this review. Since the environment directly influences biofilm formation, we also described the environmental signals that regulate the expression of functional amyloids. Understanding the regulatory mechanism of functional amyloids may help tackle biofilm-related diseases in an improvised manner and provide new avenues for drug discovery.

## 2. Biofilm Assembly and Its Gene Regulation

Biofilms are highly heterogeneous bacterial communities, where the 3D architecture and chemical composition change according to environmental conditions [2,6]. Despite heterogeneity in composition and structure, biofilm assembly is a uniform process in most bacteria [6]. The stages involved in biofilm biogenesis can be briefly described as (1) reversible attachment to the surface, (2) irreversible and stable attachment, (3) proliferation and microcolony formation, (4) maturation, and (5) dispersion [1]. Mature biofilm provides bacteria several advantages over the planktonic lifestyle and remains as one of the most featured adaptations.

Biofilm formation is regulated by various intracellular and intercellular signaling systems [25]. The major signaling systems responsible for the synthesis and assembly of various matrix constituents during biofilm formation include quorum sensing, bis-(3′-5′)-cyclic diguanosine monophosphate (c-di-GMP) signaling, and non-coding small RNAs (sRNA) [25]. Quorum sensing allows bacteria to detect cell density change through autoinducers and respond via a change in gene expression [26]. Modulation in intracellular levels of c-di-GMP leads to differential gene expression profile. Higher c-di-GMP concentration results in inhibition of motility, induce matrix-associated polysaccharide and adhesins synthesis [25]. sRNAs have been reported to regulate exopolysaccharide synthesis and export, amyloid expression, and motility [25]. 

## 3. Gene Regulation of Bacterial Functional Amyloids during Biofilm Assembly

### 3.1. Escherichia

*E. coli* express proteinaceous fibrils-like structures called curli, which are essential for cell contact and promote host colonization [15]. Curli elicit close interactions with surfaces and form inter-bacterial bundles permitting a stable cell association within the biofilm [27,28]. Chapman and his colleagues were the pioneers to provide a breakthrough in understanding the amyloid characteristics of proteins in bacteria by discovering curli in biofilms [15]. Curli is one of the best-studied bacterial functional amyloids [29]. The expression and translocation of curli are governed by **c**urli **s**pecific **g**enes (*csg*) encoded by *csgBAC* operon [30]. *csgBAC* encodes for minor curli subunit CsgB, major curli subunit CsgA, and periplasmic protein CsgC, respectively [30]. Under biofilm-forming conditions, curli assembly is initiated by CsgB that provides a suitable template for efficient CsgA amyloid assembly on the outer cell surface [31,32]. CsgC is a periplasmic protein that keeps CsgA in a soluble form within the cells [33]. Another operon *csgDEFG* encodes four accessory proteins that are essential for proper curli assembly [15]. CsgG is an outer membrane nonameric lipoprotein that aids in the secretion of CsgA and CsgB curli subunits [34]. CsgE alters CsgG pore properties and adds specificity to CsgG-dependent secretion [35,36]. Within the cells, CsgE also helps in maintaining CsgA in its soluble state [35,36]. CsgF is a curli adaptor protein that facilitates curli amyloid assembly onto the cell surface [37]. Curli biogenesis is a highly regulated process controlled by various genes and gene products [38]. The highly robust nature, striking mechanical properties, and stiffness like silk makes curli an exciting system to understand the machinery that modulates its expression [39]. Here we shed light on the significant regulators of curli expression:

CsgD: CsgD is a FixJ/LuxR transcriptional family master regulator protein that positively regulates *csg* expression [40]. The expression of CsgD is modulated at both transcriptional and post-transcriptional levels by regulatory proteins and sRNAs [41]. OmpR, RcdB, PlaR, H-NS, RstA, CpxR, and IHF are the major transcriptional factors that recognize the environmental cues and accordingly affect *csgD* expression [41]. OmpR, IH-F, RcdB, and RstA are positive regulators, while PlaR, CpxR, and H-NS are negative regulators of *csgD* expression [41]. Besides these, the complex of catabolite repressor protein and cyclic AMP also influence curli expression by positively regulating *csgD* transcription [42]. 

sRNAs: sRNAs such as McaS, OmrA, OmrB, RprA, RydC, RybB, and GcvB downregulate *csgD* expression [43,44,45]. RydC is a trans encoded sRNA that makes a complex with host factor I protein (Hfq), which is paired with transcription initiation sequences (TIS) of *csgD* mRNA [44]. The stable complex between RydC-Hfq and *csgD* mRNA hampers *csgD* expression, reducing *csgBAC* transcription, thereby drastically impairing curli biogenesis and biofilm formation [44]. RydC-Hfq mediated *csgD* downregulation is suggested to be a potential mechanism for *E. coli* to switch between planktonic and sessile state [44]. On the contrary, McaS, RprA, and GcvB interact with Hfq and RNaseE, thereby inducing ribonucleolytic cleavage of *csgD* mRNA and abolishing curli expression [45]. The OmrA/B set of sRNAs also drastically reduces *csgD* expression by inducing translational inhibition and abolishes curli synthesis [43].

Tol-Pal system: *E. coli* has a Tol-Pal system encompassing five proteins required to maintain outer membrane integrity [46] and amyloid-dependent biofilm assembly [47]. Tol-Pal cascade deactivates RcsB/A system and downregulates OmpR expression [47]. RcsB/A blocks *csgBAC* operon and inhibits CsgA expression whereas, OmpR increases *csgD* expression [47]. 

BolA-like protein family: Proteins belonging to BolA-like protein family are genetic regulators and its homologues are conserved from prokaryotes to eukaryotic organisms [48]. *E. coli* BolA (encoded by *bolA*) is a transcriptional switch and a stress regulator protein that governs a variety of phenotypes including biofilm formation, bacterial morphology, membrane permeability, and flagella formation [48,49]. Recently, BolA was found to be involved in curli formation by directly binding to *csg* operon and positively influencing its expression [49]. 

**Figure 2:**
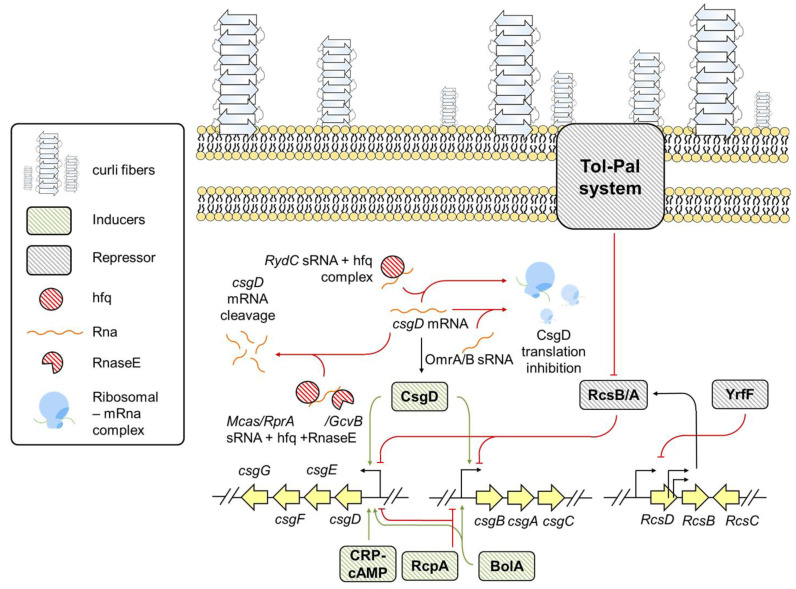
The genetic regulation of *csg* transcription in *E. coli*.

Other regulators: A recent study suggests a positive role of purine biosynthesis genes *purF*, *purD*, *purM,* and *purK* in curli expression [50]. Apart from purine biosynthesis gene, the disrupted putative membrane protein IgaA homolog; YrfF results in curli downregulation [50]. Mutated YrfF gene displays uncontrolled colanic acid production by over-expression of Rcs two-component system that negatively controls transcription of curli genes [50]. Furthermore, in the same study, a novel negative regulator of *csgD* and *csgA* transcription, named **r**epressor of **c**urli **p**roduction (RcpA) was identified [50]. As summarized in Figure 2, the post-transcriptional regulation of *csgD*, several inducers, and repressors collectively modulate *csg* expression and contribute to amyloid-associated biofilm biogenesis.

### 3.2. Bacillus 

**Figure 3:**
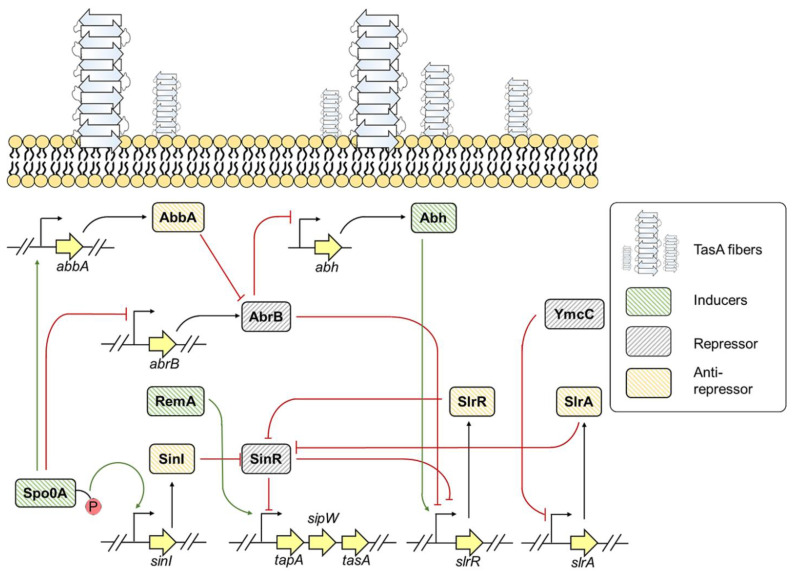
Genetic regulation of TasA expression in *B. subtilis*.

*B. subtilis* is a Gram-positive bacterium widely found in the environment [51]. It forms biofilms on solid surfaces and at the air-liquid interface [51]. The biofilm matrix of *B. subtilis* is composed of surface hydrophobic layer protein (BslA), major biofilm matrix protein component (TasA), γ-poly-DL-glutamic acid, and exopolysaccharide comprising galactose, N-acetyl-galactose, and glucose [51]. TasA is an amyloidogenic protein that readily forms amyloids under in vivo and in vitro conditions [17,52]. TasA and exopolysaccharides contribute to the architectural development and structural integrity of the biofilm [52]. **T**asA **a**nchoring or assembling **p**rotein (TapA) assists in fibril assembly of TasA onto cell membrane [53]. The amino-terminal of TasA and TapA are recognized by signal peptidase W (SipW) that trims the signal peptides during translocation of proteins to the extracellular environment [54]. The expression and secretion of TasA and TapA are under the tight control of *tapA-sipW-tasA* operon [17]. Figure 3 shows the regulation of TasA expression and the accessory proteins required for proper TasA assembly.

Sporulation transcription factor genes (Spo0A) and HTH-type transcriptional regulator (SlrR): Stage 0 Sporulation Protein A (Spo0A) is a central response regulator protein that regulates the expression of genes involved in matrix synthesis and sporulation [55]. The phosphorylated form of Spo0A (Spo0A~P) controls differential gene regulation within the cell [55]. The threshold level of Spo0A~P regulates the expression of *tapA-sipW-tasA* operon by two mechanisms [55]. First, Spo0A~P mediates the inhibition of SinR activity [55]. SinR is a master regulator of *B. subtilis* biofilm formation [55]. Under normal conditions, SinR binds to *tapA* promoter and prevents *tapA* expression [55,56]. However, in response to environmental stimuli, Spo0A~P level reaches a threshold that further induces the expression of an anti-repressor protein SinI [55]. SinI forms an irreversible heterodimer with SinR and thereby prohibits SinR from binding to *tapA* promoter, leading to *tapA* expression [55,57]. As SinI sequesters SinR, another SinR repressed gene, *slrR,* is transcribed, which further blocks SinR activity [58,59]. Thus, when SinI is expressed under the control of Spo0A~P, it inhibits SinR activity, leading to activation and accumulation of SlrR, triggering cells to produce more SlrR [58,59]. The shift from low SlrR to high SlrR state within the bacterial cells is recognized as an epigenetic switch, which helps in biofilm biogenesis for several generations *via* SinR inhibition by SlrR. Altogether, SinI and SlrR inhibit SinR activity, resulting in *tapA* expression and contribute to biofilm formation [55]. 

Secondly, Spo0A~P modulates the expression of a regulatory protein AbrB, which is a negative regulator of slrR [55]. This regulation is achieved via two mechanisms: (1) Spo0A~P directly represses the expression of *AbrB*, and (2) Spo0A~P induces expression of protein AbbA, which sequesters AbrB from slrR promoter [56,58,60,61]. In addition to slrR, AbrB also negatively regulates the abh gene, which positively regulates slrR expression [62]. Some extra-cytoplasmic function (ECF) RNA polymerase σ-factors transcriptionally regulate abh expression [63]. These factors are activated by various stress signals that suggest a regulatory process independent of Spo0A~P response regulatory mechanism [63]. 

Transcriptional regulator (SlrA): SlrA is a paralog to SinI that is involved in regulating *tapA* epigenetic switch [58,64]. Like SinI, SlrA is also a SinR repressor. Activated SlrA sequesters SinR, leaving the *slrR* promoter accessible to the transcription machinery, which leads the cell to a high-SlrR state, resulting in *tapA* expression. *slrA* expression is controlled via transcriptional repressor YmcC. However, what cues relieve YmcC from the promoter of *slrA* are yet to be explored [58,64].

Regulator of extracellular matrix (Rem): RemA and RemB are the two positive protein-regulators essential for manifesting *tapA-sipW-tasA* transcription [65,66]. RemA directly binds to the promoter region of *tapA* operon and induces the expression. However, the upstream signaling which monitors RemA activity is yet unknown [65,66]. Altogether, inducers, repressors, anti-repressors, and epigenetic switch frames a complex regulatory system that supervises *tasA* expression and contributes to TasA dependent biofilm formation.

### 3.3. Staphylococcus

Staphylococcus is a Gram-positive bacteria predominantly involved in nosocomial infections [67]. It can adhere to indwelling medical devices and adapt a biofilm lifestyle for better survival [67]. Staphylococcus biofilm matrix consists of eDNA, proteinaceous adhesins, aggregates, exopolysaccharides, and teichoic acids [67]. Two proteins, namely **p**henol **s**oluble **m**odulins (PSMs) and **b**iofilm-**a**ssociated **p**rotein (BAP), greatly influence biofilm development [18,19,68,69]. The following section will summarize the genetic control of PSMs and BAP through various regulatory mechanisms.

**Figure 4:**
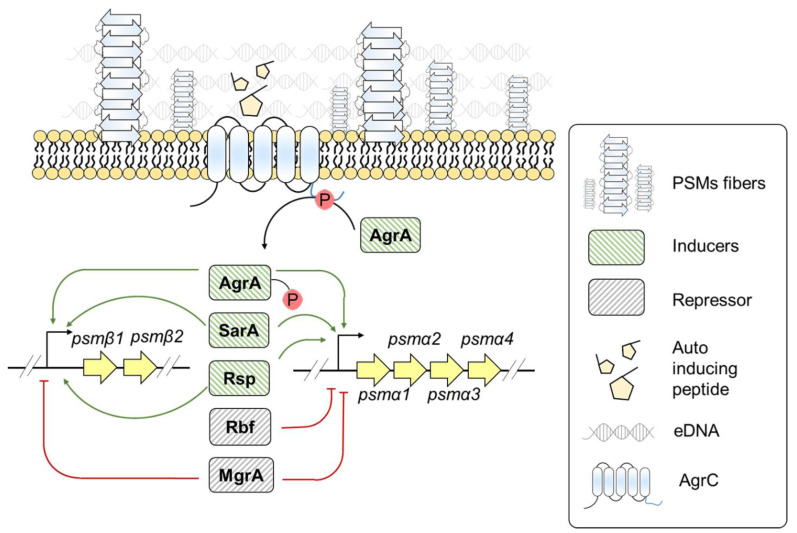
Genetic regulation of *psm* expression in *S. aureus*.

A. Phenol soluble modulins (PSMs): PSMs are small α-helical peptides involved in the virulence of *S. aureus* infections [68,70]. PSMs form amyloid-like structures and assist in biofilm stabilization by protecting the cells against matrix-degrading enzymes [18,71]. However, the monomeric form of PSMs holds relevance in biofilm disassembly [72,73,74]. There are two variants of PSMs: PSMsα and PSMβ [70]. The *psm* genes are clustered in two *loci*, expressing the shorter α type 1–4 and the longer β type 1 and 2 PSM peptides [70]. *S. aureus* PSMα1, PSMα3, PSMα4, and PSMβ1, PSMβ2 are amyloidogenic [18,75,76]. The different forms of PSMs display amyloid polymorphism with PSMα1 and PSMα4 having cross-β amyloid fibrils whereas PSMα3 forms cross-α fibrils [77]. *S. aureus* PSM fibrils involved in biofilm stabilization are majorly composed of PSMα1, PSMα2, PSMα4, and PSMβ1, PSMβ2 peptides [18]. Unlike in vitro PSM amyloid assembly, *S. aureus* grown in culture media requires eDNA for PSM polymerization [78]. The expression of PSMs is governed by different regulatory factors, as depicted in Figure 4. Here we report the major regulatory systems responsible for PSM expression:

Accessory gene regulator (Agr): The Agr system is the main regulatory system that influences the expression of several virulence factors [79]. The Agr system encompasses two divergent transcriptional units, RNAII and RNAIII [79]. RNAII encodes for AgrA-D proteins. AgrD is a precursor for an autoinducing peptide [79]. In the presence of autoinducing peptide, the sensor histidine kinase; AgrC activates AgrA that further induces RNAII, RNAIII, and *psm* gene transcription [79]. The Agr system also appears to be an important regulator for governing *psm* expression because *agr* deletion mutants result in downregulation of PSM expression [79]. RNAIII is an Agr effector molecule that downregulates the expression of genes involved in the synthesis of surface proteins and upregulates the ones responsible for exoprotein expression [80]. The role of RNAIII in cell death and subsequent increase in eDNA release suggest that the Agr system not only positively regulates PSM expression, but may create an environment that could facilitate PSM polymerization [81]. 

AraC/XylS-type regulator: The **r**egulator of **b**iofilm **f**ormation (Rbf) is an AraC/XylS type regulator modulating *Staphylococcus* biofilm formation [82]. Rbf directly represses *psmα* transcription without significantly affecting *psmβ* transcription [82]. Another AraC family regulatory protein, Rsp, positively influences *psmα* and *psmβ* transcription [83]. Rsp influences *psm* transcription in an Agr-independent manner [83]. Rsp binds to the upstream region of Agr binding site onto *psmα* operon, whereas it binds to both upstream and downstream region of Agr binding sites on *psmβ* operon [83]. The PSMs are one of the known agents for causing skin and soft tissue infections [83]. In one of the experiments using mice models, it has been demonstrated that *rsp* deletion decreases the skin abscesses in mice. However, compared to *agr* deletion mutants, *S. aureus rsp agr* double mutant failed to reduce the abscesses area [83]. Rsp seems to regulate PSM expression but may not be a critical regulator as Agr system. 

Staphylococcus accessory regulator A (SarA): SarA is another regulator that controls gene expression of virulence factors [84]. A study between foodborne and clinically associated *S. aureus* strains revealed the positive role of SarA in regulating PSM gene expression [84]. Whether SarA directly or indirectly influences *psm* expression is not yet known. Further studies are required to understand the role of SarA in PSM expression. 

HTH-type transcriptional regulator (MgrA): MgrA is a transcription regulator that affects PSM expression and biofilm formation [80,85]. It negatively regulates PSM expression by binding to *psmα* and *psmβ* operon [85]. However, *mgrA* deletion mutants display more *psmβ* expression compared to *psmα* expression [85], suggesting MgrA has more regulatory effect on *psmβ* compared to *psmα* operon. MgrA weakens biofilm detachment at the late biofilm developmental stage by repressing *psm* expression; however, its presence discourages biofilm formation at the early developmental state [85].

B. Biofilm-associated protein (Bap): Bap is a cell surface anchored protein that plays a dual role in biofilm formation [86]. The monomeric state of Bap helps in antibiotic resistance, intracellular adhesion whereas the amyloid form elevates clumping and facilitates biofilm assembly on abiotic surfaces and host tissues [86]. After secretion, Bap is covalently attached to the cell surface and processed to release the N-terminus region, which remains soluble at neutral pH, but forms extracellular amyloid-like aggregates when pH drops to the acidic range [19]. The gene encoding Bap lies within Staphylococcal Pathogenicity Island bovine 2 (SaPIbov2) [87]. SarA is the major regulatory protein that controls Bap expression. 

Staphylococcus accessory regulator (SarA): SarA directly promotes Bap-dependent *S. aureus* biofilm formation [88]. Northern blot analysis reveals reduced *bap* mRNA in the *sarA* mutant compared to wild-type [88]. Furthermore, during the late exponential to stationary phase, the expression of *sarA* is induced by its sigB (alternative sigma factor B, a stress response regulator) dependent promoter [89]. Thus, sigB may indirectly impact *bap* expression *via* regulating *sarA* expression at late exponential to stationary phase of bacterial growth [89]. 

Phase variation: phase variation is a phenomenon that drives the conversion of a non-biofilm-producing phenotype to a biofilm-producing one and vice versa [90]. This phenomenon is also observed in Bap-dependent biofilm-producing *S. aureus* strains [91]. Under in vitro conditions, *S. aureus* performs two-way conversion of phase variant phenotype, i.e., from biofilm-positive phenotype to biofilm-negative phenotype and vice versa. However, *S. aureus* infecting the sheep mammary gland displays one-way phase variant conversion, from biofilm-negative phenotype to biofilm-positive phenotype [91]. The negative biofilm phase variants of *S. aureus* have a reduced *Bap* expression, whereas the positive phase variants display higher Bap levels [91]. 

## 4. Targeting Functional Amyloids Transcriptional Regulation as an Anti-Biofilm Strategy

The biofilm community display resistance mechanisms against the conventional antibiotic through incomplete or slow permeability of antibiotics to matrix milieu, presence of different cell subpopulation, and altered chemical environment within the biofilm [92]. One way to tackle the biofilm mediated antibiotic resistance is to target the process involved in biofilm formation itself in combination with different antibiotic and/or antimicrobial compounds [93]. Here, we enlist some of the small molecules and/or natural compounds that can target genetic regulation of biofilm-associated functional amyloids (PSMs and curli) in combination with antibiotics or alone as a successful anti-biofilm strategy.

*Staphylococcus* spp.: A computational approach suggested 4-[(2,4-diflurobenzyl)amino] cyclohexanol as the best small molecule to target *Staphylococcus sarA* [94] 4-[(2,4-diflurobenzyl)amino] cyclohexanol showed anti-biofilm activity against clinically isolated multidrug resistance *S. aureus* strains, but not the anti-bacterial activity [95]. It also reduced the minimum inhibitory concentration of the antibiotic during combinational studies. Cinnamaldehyde, a major component of cinnamon essential oil present in barks and leaves of cinnamon trees, displays dose dependent anti-biofilm and antibacterial activity [96]. Cinnamaldehyde treated methicillin-resistant *Staphylococcus aureus* strains had lower levels of *sarA* mRNA [96]. As SarA positively control *bap* and *psm* expression, cinnamaldehyde and 4-[(2,4-diflurobenzyl)amino] cyclohexanol may have impact in downregulating their expression [84,88,96]. The dose dependent effect of thymol on MRSA results in MRSA biofilm inhibition [97]. However, once the dose exceeds 100 µg/mL, thymol displays antibacterial effects. Thymol mediated significant downregulation of *sarA* and sarA regulated virulent genes expression [97]. Thus, thymol have the potential to downregulate *bap* and *psm* transcription via interfering *sarA* expression. Additionally, rifampicin in combination with thymol ameliorates its antibacterial activity onto planktonic and preformed *S. aureus* biofilm cells. Moreover, 5-Dodecanolide (DD) is a phytochemical exhibiting anti-biofilm activity against MRSA and other clinical associated *S. aureus* strains. *C. elegance* treated with DD demonstrated 64% reduction in MRSA colonization compared to non-treated control [98]. DD promotes *agr*, RNAIII, PSMα, expression and downregulates *sarA* transcription [98]. DD treated cells showed elevated DNase and protease activity. Interestingly, DD decreases eDNA release in dosage dependent manner [98]. PSM in a lesser or no eDNA environment remains in a monomeric state within the culture medium. The monomeric state of PSM assist in biofilm disassembly [18,69,72,73,78,99]. Altogether, the increased expression of PSM in a lesser eDNA environment along with elevated protease activity could be a possible mechanism of DD to exhibit anti-biofilm activity.

*Escherichia* spp.: Epigallocatechin gallate (EGCG) is a green tea polyphenol that has been shown to inhibit *E. coli* biofilm formation and possess antibacterial activity at a much higher concentration [100]. EGCG inhibits curli expression and amyloid formation *via* reduced expression of *csgD* in *E. coli* cells [100]. Curli has been known to play an important role in early progression of Parkinson’s disease in α- synuclein overexpressing mice [101] by accelerating α-synuclein amyloid formation. However, the presence of EGCG not only inhibits curli mediated amyloid formation but also improves motor impairment in α-synuclein overexpressing mice [101]. Likewise, EGCG inhibits amyloid formation by human proteins such as transthyretin (TTR), α-synuclein and amyloid-β peptide [102,103,104,105].

Thus, EGCG is a potential candidate for inhibition of amyloid-dependent biofilm formation. Another phenolic compound named ginkgolic acids from *G. biloba* profoundly inhibits Enterohemorrhagic *E. coli* O157:H7 (EHEC) biofilm formation [106,107]. Similarly, coumarin and umbelliferone have anti-biofilm effect on *E. coli* O157:H7 strain [108]. Furthermore, coumarin also modulates motility, quorum sensing and toxin related gene expression. In another study, 83 essential oils for inhibiting Enterohemorrhagic *E. coli* O157:H7 (EHEC) biofilm were evaluated out of which pimento berry, clove, cinnamon bark, and bay oil gave the best result, reducing more than 75% of the biofilms [109]. Further analysis suggested the eugenols to be the essential components for its anti-biofilm activity of the oils [109]. In an animal model of *C. elegans* infected with EHEC, it was observed that the worms survived when treated with clove or eugenols as compared to non-treated controls [109]. Phloretin, an antioxidant present in apples, demonstrate anti-biofilm activity against *E. coli* O157:H7. Phloretin repress autoinducer-2, curli and toxic gene expression [110]. In a dose dependent manner, the honey from different floral sources such as clover, acacia and polyfloral display *E. coli* O157:H7 biofilm inhibition. Along with downregulation of *csg* gene, honey reduces quorum sensing and virulence gene expression within *E. coli* O157:H7 [111]. Altogether, different kinds of chemicals have been explored to target the genetic regulators involved in functional amyloid regulation. 

## 5. Environmental Factor Regulating Gene Expression of Biofilm-associated Amyloids

The bacterial cells sense and respond to environmental cues *via* altered gene expression. Modulation in gene expression results in a change of protein pool within the cytoplasmic milieu that governs the formation or dissociation of the biofilm matrix [25]. Therefore, it is of prime interest to study the environmental factors that affect the regulation of matrix components. Here, we discuss some of the environmental factors that regulate the expression of biofilm-associated amyloids.

## 6. Environmental Factors Influencing *csg* Expression in *E. coli*

**Figure 5:**
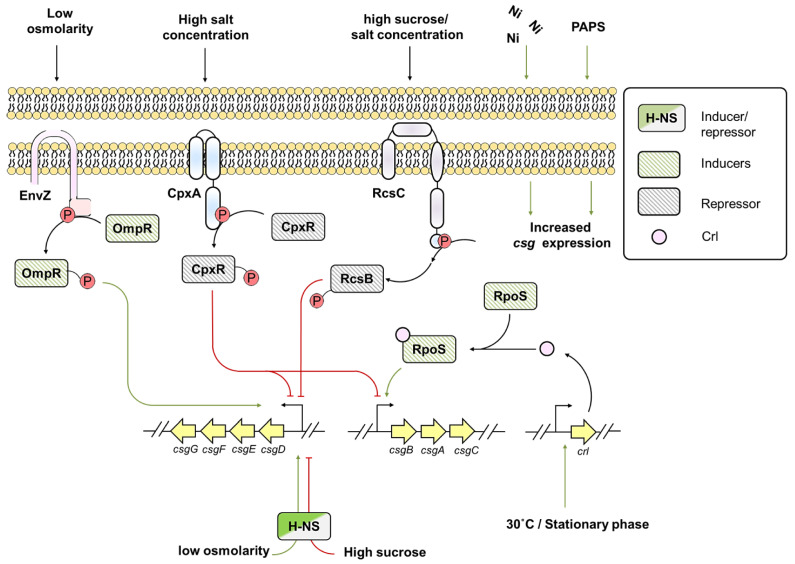
Interplay between various environmental factors that regulates *csg* transcription.

The adequate expression of curli depends on several environmental factors such as altered osmolarity, low temperature, and stationary growth phase conditions, as shown in Figure 5 [112,113,114]. During low osmolarity conditions, EnvZ/OmpR two-component system and histone like nucleoid structuring (H-NS) protein drive *csg* expression [115]. EnvZ is a sensory kinase of EnvZ/OmpR two-component system that senses osmolarity change and phosphorylates OmpR, which positively regulates *csgD* expression [114,116]. However, at low osmolarity and high salt condition or in the presence of high sucrose concentration, H-NS represses *csgD* expression [114]. CpxA/CpxR two-component system also influences *csg* expression [114]. CpxA possesses kinase phosphatase activity, whereas CpxR is a response regulator of a two-component system. Under physiological conditions, more phosphatase and less kinase activity of CpxA onto response regulator CpxR is observed, leading to de-repressed activity of the downstream targets [112]. Whereas in high salt concentration, phosphorylated CpxR hampers *csgD* expression thereby reducing *csgA* transcription [112,114]. Besides, at high sucrose or salt concentration, RcsC sensory kinase from Rcs two-component system phosphorylates RcsB contributing to *csg* downregulation [114,117,118].

Temperature is another major environmental factor that regulates curli expression [113]. Some strains of *E. coli* synthesize curli at low temperature (~30 °C), which is accomplished by a small protein called Crl [113], levels of which are elevated during the transition to stationary phase [113]. Crl forms holoenzyme with the alternative sigma factor σ^s^ (RpoS) and activates *csgBA* promoter [113]. Natural F plasmids in *E. coli* strains lead to the curli biogenesis by upregulating *csgBAC* operon at 37 °C [119]. Altogether, a thermosensing mechanism allows *csgA* expression at various temperatures [113].

The presence of metals also enhances curli expression [120]. Exposure to sub-inhibitory level of nickel leads to high expression of curli and biofilm thickening [120]. Similarly, sulfur is required for *E. coli* surface adhesion and biofilm formation. Sulfate is the primary source of sulfur as it is abundant in the environment. Once taken up by the bacterium, sulfate is reduced to hydrogen sulfide *via* an assimilation pathway [121] that results in formation of two nucleotides: adenosine 5′-phosphosulfate (APS) and phosphoadenosine 5′-phosphosulfate (PAPS) [122]. The inactivation of PAPS reductase coding gene *cysH* leads to the overproduction of PAPS in the medium, which promotes curli production [121]. Along with metals, antioxidants, such as vitamin C in its minimum inhibitory concentration, inhibits biofilm formation by *E. coli.* Vitamin C affects the quorum sensing activity and exopolysaccharide production, thereby interfering with downregulation of genes responsible for biofilm formation such as *csgA, csgG, and fimA* [123]. 

## 7. Environmental Factors Regulating *TasA* Expression in *B. subtilis*

*B. subtilis* fends against various environmental stresses such as osmotic pressure, nutrient availability, and bactericidal agents [124,125]. The osmotic pressure created either by the addition of polyethylene glycol (PEG) or due to the presence of exopolysaccharide results in downregulation of *tapA-sipW-tasA* operon, thereby inhibiting gene expression of matrix components [124]. The inhibitory effect is SinR and KinD dependent [124]. KinD is a phosphotransferase, being part of the network that can phosphorylate Spo0A [124]. Threshold levels of Spo0A~P facilitated *tasA* expression [55,57]. Elevated Spo0A~P levels are required to stimulate the significant expression of sporulation specific gene *SpoAII*. Besides, the presence of PEG leads to KinD dependent *SpoIIA* expression [124]. Interestingly, *B. subtilis* need to bypass the matrix formation to achieve a sporulation state which is also KinD dependent [126]. Altogether, higher osmotic pressure will elevate Spo0A~P levels that may bypass the matrix formation, downregulating *tasA* expression, and aid in sporulation [124,126].

**Figure 6:**
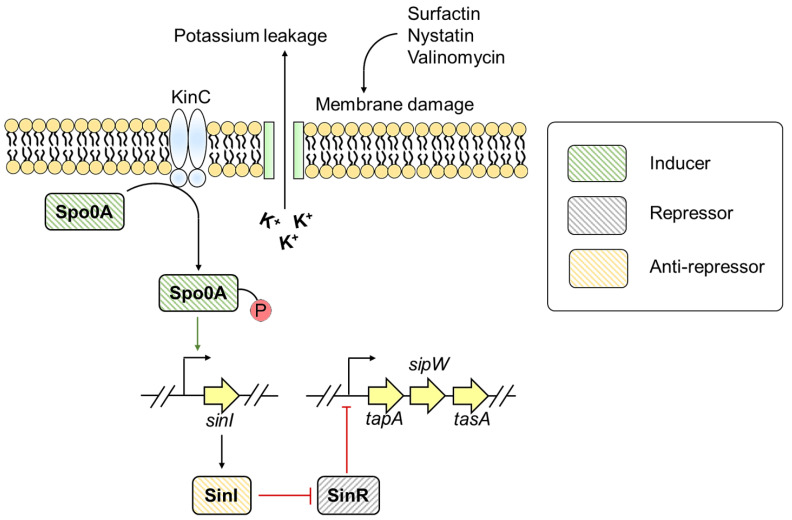
Role of antibiotics and antifungal in modulating *TasA* expression.

Molecules and metabolites such as nystatin from Streptomyces and auto-inducers like surfactin can stimulate biofilm formation in *Bacillus* [125]. Surfactin, nystatin, and valinomycin can disrupt cell membrane leading to potassium leakage [125]. The potassium leakage is sensed by Sporulation kinase C (KinC) that phosphorylates Spo0A resulting in Spo0A~P production. When Spo0A~P reaches a threshold level, the sub-population produces a biofilm matrix *via* stimulating *tasA* operon (Figure 6) [125]. This gives an insight into how *Bacillus* spp. establishes communication with other microbes and colonize in unfavorable environmental conditions [125]. 

Under nutritional stress, *B. subtilis* displays cannibalism that delays or avoids entry into the sporulation phase [125]. Certain bacteriocins, such as surfactin, trigger the production of cannibal toxins (sporulation killing factor, sporulation delaying protein) and matrix components like TasA [125] that favors the growth of matrix producers. In contrast, the cells that are unable to transcribe matrix-producing genes are lysed. The lysed cells secrete a set of nutrients on which matrix producers feed, grow, and delay the entry into the sporulation phase [125].

## 8. Conclusions

Biofilm assembly is a highly regulated process with various genes playing a pivotal role in the synthesis and organization of matrix components. Amyloids, being a robust scaffold, contribute significantly to the architecture of the majority of bacterial biofilms. Dedicated systems and genes strictly regulate the amyloid assembly during biofilm formation. Bacterial amyloid acts as a double-edged sword as it provides structural–functional aspects to the biofilm and contributes to the manifestation of numerous infectious diseases. Therefore, understanding the genetic control of amyloids will help us target the genes involved in its regulatory mechanism and pave the way to curb amyloid-associated biofilm infections.

## Data Availability

No new data were created or analyzed in this study. Data sharing is not applicable to this article.
